# An image fusion study of the geometric accuracy of magnetic resonance imaging with the Leksell stereotactic localization system^1^

**DOI:** 10.1120/jacmp.v2i1.2627

**Published:** 2001-01-01

**Authors:** Cheng Yu, Zbigniew Petrovich, Michael L. J. Apuzzo, Gary Luxton

**Affiliations:** ^1^ Department of Radiation Oncology (CY, ZP), Department of Neurosurgery (MLJA) University of Southern California, Keck School of Medicine 1441 Eastlake Avenue Los Angeles California 90033; ^2^ Department of Radiation Oncology Stanford University School of Medicine Stanford California 94305

**Keywords:** radiosurgery, image distortion, geometric accuracy

## Abstract

A special acrylic phantom designed for both magnetic resonance imaging (MRI) and computed tomography (CT) was used to assess the geometric accuracy of MRI‐based stereotactic localization with the Leksell stereotactic head frame and localizer system. The acrylic phantom was constructed in the shape of a cube, 164 mm in each dimension, with three perpendicular arrays of solid acrylic rods, 5 mm in diameter and spaced 30 mm apart within the phantom. Images from two MR scanners and a CT scanner were obtained with the same Leksell head frame placement. Using image fusion provided by the Leksell GammaPlan (LGP) software, the coordinates of the intraphantom rod positions from two MRI scanners were compared to that of CT imaging. The geometric accuracy of MR images from the Siemens scanner was greatly improved after the implementation of a special software patch provided by the manufacturer. In general, much better accuracy was achieved in the transverse plane where images were acquired. Most distortion was found around the periphery while least distortion was present in the middle and most other parts of the phantom. For most intracranial lesions undergoing stereotactic radiosurgery, accuracy of target localization can be achieved within size of a voxel, especially with the Siemens scanner. However, extra caution should be taken for imaging of peripheral lesions where the distortion is the greatest.

PACS number(s): 87.61.–c, 87.57.–s

## INTRODUCTION

In stereotactic radiosurgery, a single or multiple fraction high dose of radiation is accurately delivered to an intracranial lesion, which is defined by an extracranial reference system, such as the Leksell and the Brown‐Roberts‐Well (BRW) head frame and localizer systems.^1^ ^–^ ^9^ Different imaging modalities, such as MRI, CT, and angiography, are currently used for target localization in stereotactic radiosurgery.^10^ ^–^ ^15^ In general, CT images provide a precise geometric localization with less contrast resolution of the lesion and its surrounding anatomic structure.^16^ On the other hand, many radiosurgical targets are better visualized with MRI as a result of superior contrast than with CT. In addition, MR images are more susceptible to spatial distortions caused by several factors, which include inhomogeneities of the constant magnetic field, nonlinearity of the gradient field, linear scale error, instrument imperfections, magnetic susceptibility artifacts, and local magnetization effects.^15^ ^,^ ^17^ ^–^ ^25^

In 1992, Kondziolka *et al*.^13^ compared the accuracy of stereotactic coordinates determined by MRI and CT studies in 41 patients with 53 targets being treated. By measuring the coordinates in each plane and as vector distances between the target and the center of the stereotactic frame on axial or coronal MRI studies, they found that the mean difference in measurement in the *x* axis was 1.19 mm and 1.55 mm in the *y* axis. Similar average differences between CT and MRI coordinates were also reported by Bednarz *et al*. in their Radionics skull phantom, the Rando head phantom, and 11 patients study.^26^ In an MR phantom study, Walton *et al*.^27^ reported much larger errors, up to 2.7 mm for the *x* axis, 7.0 mm for the *y* axis, and 8.0 mm for the *z* axis. They also found that more accurate stereotactic localization could be achieved with a three‐dimensional image acquisition.^28^

More recently, Orth *et al*.^29^ reported a study on the geometric accuracy of the MR image for stereotactic localization with the Brown‐Roberts‐Wells (BRW) head frame system by manually fusing MR data set with CT images. They found that differences in fiducial marker positions (up to 1.99 mm in anteroposterior or the *y* direction) were correlated with differences in intraphantom target positions (up to 1.97 mm in the same direction) and suggested that improper fiducial rod identification and the subsequent transformation to stereotactic coordinate space were the greatest sources of spatial uncertainty.

In this study, a special acrylic phantom designed for both MRI and CT scanners was constructed to evaluate the geometric accuracy of MR imaging for the Leksell stereotactic localization by fusing CT and MRI data sets together in a series of transverse, coronal, and sagittal images, using CT as a standard.

## MATERIALS AND METHODS

### Phantom

**Figure 1:**
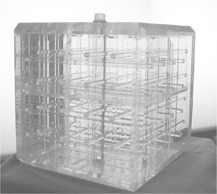
Photograph of the phantom.

The phantom was precisely machined and constructed as a cube with an outer dimension of 164 mm, as seen in Fig. 1. The phantom contains three perpendicular arrays of solid acrylic (polymethylmethacrylate, PMMA) rod, 5 mm in diameter, spaced 30 mm apart between the nearest rods in the same array, and either 20 mm, or 30 mm away from the inner surface of the phantom. All the rods as well as the edges of each plate can be used as markers for the measurement of the stereotactic localization. There were 16 rods in each array or plane. One of the 48 rods was replaced with a 16‐mm acrylic tube for a future investigation, such as inserting a foreign object into the body of the phantom. The thickness of the six plates for the phantom was 12.7 mm, which was felt to be strong enough to keep all the rods and plates in their correct positions under a pressure of the Leksell head frame placement. Three additional 3‐mm thickness plates were used as dividers for the enforcement of the rod positioning. The dividers were located approximately at the middle of the phantom and were parallel to its relative sides and perpendicular to each other. The phantom with an inner dimension of 140 mm was filled with the cupric sulfate ${(\text{CuSO}}_{4}\cdot {5H}_{2}O)$ solution of approximately 0.001 Mol/L to enhance the image of the rods from both CT and MRI scanning.

### Leksell head frame

The Leksell stereotactic head frame was attached to the phantom by using four aluminum posts and four aluminum fixation screws with a metal tip. The stereotactic head frame was placed in such a fashion that the center of the phantom was virtually coincident with the center of the head frame. The head frame was made of lightweight aluminum and had four feet that connect it to the angiography, CT or MR adapters. Heavy metals have been removed from the Leksell frame to reduce the probability of artifacts in CT imaging. The frame is also devoid of magnetic materials allowing it to be used in conjunction with MRI.

### Image acquisition

MRI and CT data sets of the phantom were obtained with the same head frame placement at the imaging facility of the University of Southern California University Hospital. A complete CT image data set (70 slices, 512×512 pixel matrix) with a 2‐mm thickness and zero gap in the transverse plane was acquired for the whole phantom with a Philips Tomoscan SR 7000 CT scanner (Philips Medical System, Shelton, CT). The field view was $300\times 300{\text{mm}}^{2}$. The image data set was first transferred through the hospital network system to a DICOM box and then to the Leksell GammaPlan software system (Elekta, Norcross, GA).

The MRI acquisitions were performed on the same day with the use of two different imaging scanners. One was a Philips Gyroscan, 1.5T MRI scanner (Philips Medical System, Shelton, CT), installed in 1991. The other one was a Siemens Symphony, 1.5T MRI scanner (Siemens, New York, NY), installed at the end of 1999. One set of $T_{1}$‐weighted transverse sequences (70 slices, 256×256 pixel matrix) with a 2‐mm thickness and zero gap for the whole phantom was acquired for each MR scanner. The field of view was $260\times 260{\text{mm}}^{2}$ for both MR scanners. The repetition time was 670 ms for the Siemens and 620 ms for the Philips scanner. The echo time was 15 and 20 ms for Siemens and Philips, respectively. A special software patch, or large field‐of‐view (FOV) remapping was implemented by Siemens for improving image spatial accuracy, specifically for stereotactic radiosurgery. The software provides a large field of view remapping, or correcting for distortion caused by the non‐linear gradient field. FOV in the new software was reduced to 180 mm from 400 mm. All image data sets were directly transferred through the hospital network to the Leksell GammaPlan (LGP version 5.20, Elekta, Norcross, GA) software system on a computer workstation.

### Image fusion

Based on information of the fiducial markers from all images in each data set, the pixels in each image were registered to the coordinate system of the Leksell head frame. After being converted to the Leksell stereotactic coordinate system, the image set can be reviewed separately or collectively in three principal planes, namely the transverse, coronal, and sagittal plane. Upon completion of the image registration, the software reports the mean and maximum errors of the fiducial markers. The errors are an estimate of the accuracy of fiducial marker correlation through the entire image study. The image fusion tool provided by the software blends any two defined studies together so that anatomical structures can be clearly visualized by enhancing the best features of both studies. In this investigation, the image data set from each MR scanner was separately fused to the CT image data set. This was performed by setting maximum or minimum gray scale values, by mixing the studies, or by subtracting the characteristics of one study from the other. It was assumed that the CT images were accurate and precise. The discrepancies in the rod positions between the MRI and CT data sets were measured, to determine the MRI distortion, or localization uncertainty.

## RESULTS AND DISCUSSION

**Figure 2:**
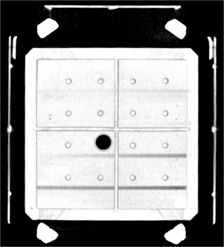
Fused image data set in the transverse plane from the Philips CT and MR scanners. A group of three fiducial markers was shown at the right lateral, the left lateral and the anterior location. The four trapezoidlike white objects were images of the Leksell head frame posts. The white circles inside the square shape of the phantom were rod images from the CT data set, the dark circles, or shadows around the white circles for the MRI. The big dark circle near the center of the phantom is the image of the tube. The cross image (mostly white) at the center presents the cross section of the divider. The horizontal lines were images or partial images from another perpendicular rod array.

Figure 2 shows results of fused phantom images with fiducial markers in the transverse plane. For this particular view, the *z* coordinate was 100 mm, or near the middle of the Leksell stereotactic coordinate system. Images of the rods and fiducials acquired from the Philips MR scanner were matched very well with those obtained from the CT scanner. However, discrepancies were noted for MR images from the Siemens MR scanner before the implementation of software patching, as compared to the CT images. These discrepancies appeared around the periphery of the phantom for the rod and at the right side for the fiducials.

One of the sources of localization uncertainty was the misregistration of the fiducial markers. The errors in determination of the fiducial position as calculated by the LGP software were very similar for both MR scanners with a mean of about 0.6 mm and a maximum of 1.4 mm in the transverse plane, both of which were slightly larger than those of CT (mean: 0.5 mm, maximum: 1.1 mm). The maximum errors were normally located around the periphery and near the aluminum fixation pins that fixed the phantom to the head frame. This effect was probably due to the combination of the MR image distortion caused by the scanner itself and a small amount of metal at the tip of the pin. The coronal or sagittal plane images would be expected to have larger image distortion, as compared to the transverse or axial plane images. For the image registration, the anterior fiducial markers, or the third plate in the Leksell stereotactic localizer, were not used in this study. It was found in our study and also by others^27^ that the errors in the fiducial coordinate were generally larger when the anterior fiducial markers were included in the process of the image registration. Generally larger distortion on the periphery could significantly effect the spatial accuracy of images due to the fact that an error in the fiducial marker position results in a misregistration of the whole image when it is transferred into the Leksell coordinate system. In our clinical practice, the third plate was always turned off when images were transformed into stereotactic space.

In this study, the assessment of the geometric accuracy of MR images was based on the results from either the original 70 slices in transverse plane of 2 mm thickness with zero slice gap through out the entire phantom or the reconstructed images in the coronal or sagittal plane from the transverse plane slices. A rod from one of three rod arrays would appear as an image of a dot or circle in its corresponding plane of view. The maximum absolute errors were obtained by evaluating the maximum displacement among all rods or datum point pairs (15 in transverse plane, and 16 in sagittal and coronal planes) in each image slice. Points for the maximum absolute errors occurred near the periphery of the phantom. The averaged deviation of all rods in each image slice was significantly smaller than that of the maximum deviation.

### Philips MR scanner

**Figure 3:**
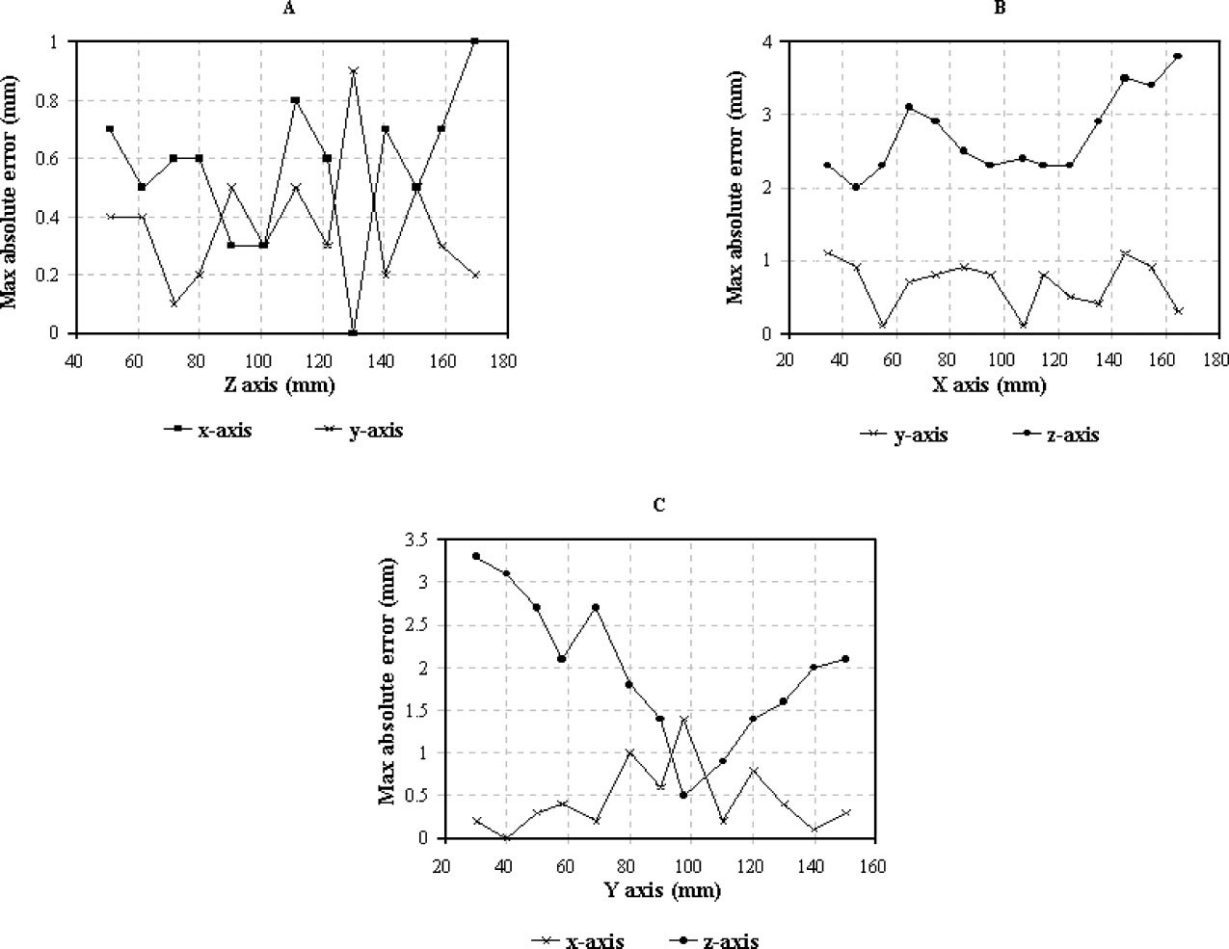
Maximum errors of the MR image for the Philips MR scanner from the image fusion study. (a) axial image, (b) reconstructed sagittal image, and (c) reconstructed coronal image.

Figures 3(a) and 3(b) show the maximum deviations of the MR images from the Philips scanner in the stereotactic coordinate system as compared to that of CT images. The sagittal images were reconstructed from the $70T_{1}$‐weighted transverse images with 2‐mm thickness and zero gap across the entire phantom. There were 15 rods in the axial plane and 16 rods in the other two planes. For the axial plane [Fig. 3(a)], the maximum absolute errors ranged from 0.0 mm to 1.0 mm with a mean of 0.6 mm in the *x* axis and from 0.1 to 0.9 mm with a mean of 0.4 mm in the *y* axis. The deviations or errors in both *x* and *y* coordinates were virtually flat in the direction of the *z* coordinate across the entire phantom. The mean values of the maximum errors were well within the uncertainty of the pixel size (approximately 1 mm×1 mm).

For the reconstructed sagittal view [Fig. 3(b)], the maximum errors were similar to those in the transverse view and ranged from 0.1 mm to 1.1 mm with a mean of 0.7 mm in the *y* coordinate. In contrast, the maximum errors in the *z* coordinate were significantly worse throughout the entire phantom region and deteriorated as the *x* axis approached larger values, corresponding to the left side in the supine position. The mean value of the maximum errors was 2.7 mm with a range of 2.0 mm to 3.8 mm. This mean value is substantially larger than the thickness (2 mm) of the original transverse image. For the reconstructed coronal view [Fig. 3(c)], the maximum errors in the *x* axis ranged from 0.0 mm to 1.4 mm with a mean of 0.4 mm, slightly larger than the results obtained from the axial view. On the other hand, the mean value of the maximum errors in the *z* axis was 2.0 mm with a range of 0.5 to 3.3 mm. The maximum errors increased as the *y* coordinate approached two ends, or the anterior (larger *y* values) and the posterior (smaller *y* values). The most distortion of the image occurred at the left‐posterior‐inferior corner of the phantom. The mean of the maximum deviations of all rods in each image slice ranged from 0.9 mm to 1.5 mm in the *z* coordinate.

MR images from the Philips scanner provided the spatial accuracy within size of a pixel (1 mm×1 mm) in the transverse plane (i.e., *x* and *y* coordinates) throughout the entire phantom as compared with CT images. On the other hand, much larger deviations, greater than the image thickness (2 mm), in the *z* coordinate were observed from the reconstructed sagittal or coronal images. These deviations, however, occurred almost exclusively around the periphery of the phantom. In the other words, the quality of the image in the transverse plane was better in the middle of the phantom.

### Siemens MR scanner

**Figure 4:**
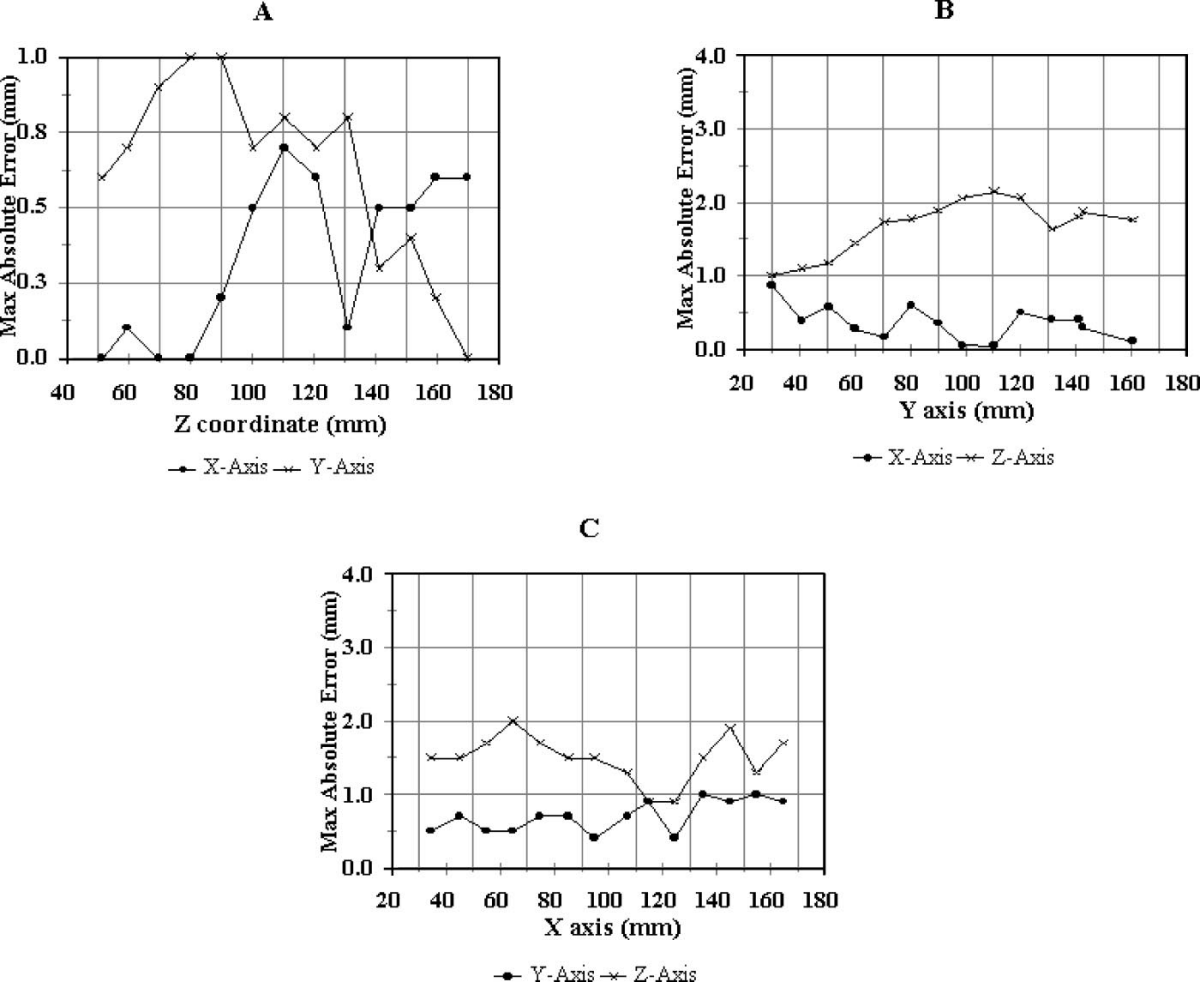
Maximum errors of the MR image for the Siemens MR scanner from the image fusion study. (a) axial image, (b) reconstructed coronal image, and (c) reconstructed sagittal image.

The results of the Siemens MR image fused with CT study were somewhat similar to those of the Philips MR and CT image fusion. However, there were differences in the geometric accuracy of MR images between the two scanners used in this study. The initial image fusion studies revealed much greater image distortions. The mean values of the maximum errors were 0.8 mm (range: 0.2–1.4 mm) for *x*, 1.5 mm (range: 0.9–2.3 mm) for *y*, and 4.0 mm (range: 2.0–5.5 mm) for the *z* axis. After complaining to the manufacturer, new patching software was then implemented specifically for stereotactic radiosurgery by the Siemens to improve image spatial accuracy. Figures 4(a), 4(b), and 4(c) show the maximum errors of the MR images after the special software patch as compared to CT in the Leksell stereotactic coordinate system. The coronal and sagittal images in Figs. 4(b) and 4(c) were reconstructed from the $70T_{1}$‐weighted axial images with a 2‐mm thickness and a zero gap. Each pair of points represented the maximum absolute error of rod coordinates for each image slice deviated from the coordinates measured from CT image. The maximum absolute errors were calculated from 15 rods in the axial plane and from 16 rods in the sagittal and coronal planes. The mean values of the maximum error for the axial plane were 0.3 mm with a range of 0.0 to 0.7 mm in the *x* axis and 0.6 mm with a range of 0.0 to 1.0 mm in the *y* axis, as seen in Fig. 4(a). The maximum errors for the *x* axis were generally smaller than these for the *y* axis, except for z>140 mm.

For the reconstructed coronal view, as seen in Fig. 4(b), the mean values of the maximum error were 0.5 mm with a range of 0.0 mm to 0.9 mm for the *y* coordinate, and 1.6 mm with a range of 1.0 mm to 2.1 mm for the *z* coordinate. The maximum errors in the *y* axis were very similar to the results obtained from the original axial images. The slightly larger maximum errors in the *z* axis occurred at z≥90 mm, i.e., from the middle to the bottom of the phantom. The results from the reconstructed sagittal view [Fig. 4(c)] were similar to those of the reconstructed coronal images.

At the current conditions or specifications in which the MR scanners were maintained during this investigation, the Siemens Symphony MR scanner provided a higher accuracy of the target localization when implemented with the special software patch as compared with the Philips Gyroscan, using the CT image as a standard. The worst errors occurred near the bottom of the Leksell head frame. The smallest distortion appeared around the region where *z* is about 100, or around the middle of the head frame. In addition, the *x* and *y* coordinates in the axial plane were in good agreement with CT images. However, the deviation in the *z* axis determined from other two planes (i.e., sagittal and coronal) was significantly larger as compared to the other two coordinates (i.e., *x* and *y*). Due to the fact that only one image scan was used in this study for each MR scanner, the effect of setup error and daily variation of imaging parameters was not taken into account.

Image fusion, using the CT as a standard, is a useful tool in quality assessment of MR imaging stereotactic target localization if the accuracy of the CT image has been verified. In our center, periodic quality checks of MR images were performed based on the results from the image fusion study using the CT as a standard. In addition, special attention was taken to the region where the errors in the *z* coordinate were the greatest.

## CONCLUSIONS

The quality of MR images in the spatial accuracy varies from scanner to scanner even with the same scanner running under different conditions. Periodic and comprehensive accuracy assessments of MR images in the stereotactic target localization should be implemented in the quality assurance for the stand‐alone MRI‐based stereotactic procedure. For a particular image scanner, the accuracy of MR images for the stereotactic localization may vary from slice to slice. For most of intracranial lesions undergoing Leksell radiosurgery procedure, the accuracy of the target position can be achieved within size of a voxel, especially for the Siemens scanner with the special patching software. However, extra caution should be taken in the case of peripheral lesions where the distortion is generally the greatest. The characteristic of MRI image distortion obtained from this study strictly applies to the specific scanner, the specific fiducial system, and an assumption of accurate CT images.
